# Confusion Worse Confounded

**Published:** 1907-02

**Authors:** 


Confusion Worse Confounded.—There is an element in our State Medical Association who favor mixing up with the pathies, recognizing them and putting them on a common footing with the regular profession; absorbing them, in fact, in pursuance of the heresy preached by the peripatetic McCormack at the behest of The Octopus. This element "tried it on" at Fort Worth last April, it will be remembered. A bill for a mixed board of medical examiners was prepared in which recognition was given to all the so-called "schools," so many of each to be on the board. This bill was kept up the sleeve of its authors, to be sprung on the House of Delegates; that is, it was not published by the association journal. But the "Red Back" got hold of it and gave it away. The *State Journal of Medicine* complained that the "Red Back" had "scooped" it; anticipated it in "giving to the profession this important piece of news," the editor wrote. In so doing I rendered, as I thought, a valuable service, prevented what I thought would be a stultification. By the time the Association met in April such opposition to the proposed mixed bill had been worked up, that to avoid a contest and a division of the Association, those having the monstrosity in hand thought best to suppress it, and it was never presented, as written.

But, instead, the bill as approved then (and as it appeared in the Association's journal for January), went through without opposition. It provides for a board of eleven physicians, to be appointed by the Governor, without naming any "schools" and without suggestion or dictation from any State association.

Well, this bill was introduced in the Senate by Mr. Looney and referred to the committee of which he is chairman. The pathies pawed the earth and fought desperately for recognition. The bill was reported favorably without alteration.

Meantime the original bill was introduced in the House, went to the committee, where the fight was renewed and all of the old straw was thrashed over. The pathies won out! There were present some of the State Association's committee and several volunteer physicians, amongst the best known members, and I grieve to say—they compounded with the pathies; agreed to the monstrous proposition that a board of ten shall be appointed, on which five "schools" of medicine shall be represented, and that no one "school" shall have a majority. That is, the regular profession (the State Medical Association, with its 3000 members) shall have *five,* and the homos, the eclectics, the physio-meds and the osteos shall have five, representing, I understand, an aggregate of less than 500. A pro-rate apportionment would give each pathy about one-tenth of a member.

Now, who is responsible for this? Who was authorized to undo what the Association did at Fort Worth?—practically restore the status of the mixed board bill which was so emphatically repudiated at Fort Worth by the House of Delegates?

Moreover, I understand that these gentlemen compounded with the Christian Science people; agreed to exempt them on certain conditions. As they are already exempt under the existing law—this was, in my judgment, sensible and right. It will be remembered that I was censured and criticised by certain 2x4 members for advocating this course before the Twenty-ninth Legislature. Tie their hands; prohibit them practicing surgery, obstetrics and on contagious diseases, and let them alone.

Thus the House and the Senate are at cross purposes. The Senate having refused to compound with the pathies, but approved the straight bill, leaving the selection to the Governor, will never accept the House amendments; and the House having been advised by representatives of the State Medical Association to mix up things with the quacks, will not accept the Senate bill. Here's a "pretty how-de-do." It will necessitate a joint conference, and no man can foresee the result—if it ever "results." *Facilis averni descendens.*

The current in the State Association has set in so strongly quackwards, that it seems futile to endeavor to stem it; but I would be false to the best traditions of the profession, false to my ideals and to my whole course as a medical journalist were I to fail here and now to go on record, for the hundredth time, as protesting, with all my soul, against the monstrous proposition to absorb the quacks and to ask for or consent to affiliation with them. Let them drop their trade marks and be examined, if they call themselves "physicians."

---

THE APPOINTMENTS to the medical offices of the State so far as they have been announced to date (February 15) are:

State Health Officer, Dr. W. M. Brumby of Houston, vice Tabor. (By the bye, Dr. Tabor is, under the new Federal law regulating the militia, still Surgeon General, and the State Health Officer is no longer ex-officio Surgeon General.)

Assistant to State Health Officer, Dr. Holman Taylor of Marshall.

Superintendent State Lunatic Asylum and Pasteur Institute (Austin), Dr. B. M. Worsham (re-appointed).

Superintendent North Texas Insane Asylum at Terrell, Dr. Gregory of Gilmer.

Superintendent Southwestern Insane Asylum, San Antonio, Dr. W. L. Barker of San Antonio, vice Dr. T. O. Maxwell. Assistant physicians, Dr. E. H. Gray, J. A. McIntosh and Bruce Allison.

Superintendent Epileptic Colony, Abilene, Dr. Jno. Preston (re-appointed). Dr. Preston launched this great institution and his administration for the four years has been a brilliant success. It is now on a fine footing.

The Quarantine Inspectors are as follows, all twelve-months men: (No appointment has been made or will be made at the yellow fever, six-months stations, towit: Velasco, Pass Cavallo and Aransas Pass.)

Inspector at Galveston, Dr. J. P. Tucker of Houston (formerly of Overton), vice McClendon.

Inspector at Sabine Pass, Dr. J. H. Florence of Dallas, vice W. R. P. Thompson. Dr. Florence succeeded the lamented Wolff at Brownsville last year.

Inspector at Brownsville, Dr. V. P. Armstrong of Dallas, vice Florence, transferred.

Inspector on the Rio Grande-Mexican border, El Paso, Dr. P. J. Shaver (re-appointed).

Inspector at Eagle Pass, Dr. Van E. McFarland, vice Dr. I. H. Brewton.

Inspector at Laredo, Dr. W. E. Lowry, vice McKnight.

Should the Legislature ratify the act of Congress, turning over international quarantine to the Marine Hospital Service of the United States Public Health Department, selling the State quarantine property to the United States, these officers will probably—should be—abolished, as it would look ridiculous to continue a State quarantine line side by side with a national quarantine service. The money for such useless and unnecessary inspection can be better used.

